# Supplementary material for: Investigating the impact of database choice on the accuracy of metagenomic read classification for the rumen microbiome
Source: Anim Microbiome. 2022 Nov 18;4:57. doi: 10.1186/s42523-022-00207-7 (PMC9673341; doi:10.1186/s42523-022-00207-7)
Supplement: Supplementary file 1 — Additional file 1: Table S1. Classification rate of reads for six reference databases at various taxonomic levels. Classification rate refers to whether the read was classified, or unclassified, regardless of accuracy. Each row denotes the six databases used to classify reads with Kraken2. The “Overall” column refers to the percentage of reads that were classified or unclassified by Kraken2 regardless of taxonomic level. Subsequent columns refer to the percentage of reads that were classified or unclassified by Kraken2 at various taxonomic levels as shown in the column headers. Table S2. Classification status of reads compared to the ground truth for the six reference databases at various taxonomic levels. The databases and detailed classification status are shown in the first column. Subsequent columns contain the percentage of reads at that taxonomic level, which had been classified by the database and had the particular classification status outlined in the first column. “Correct” and “incorrect” refer to reads that were classified correctly or incorrectly by Kraken2 using the respective database. “Truth unknown” refers to the reads that originate from genomes that do not have an assigned family or genus. “Unclassified at any level” refers to reads that were not classified to any taxonomic level. “Unclassified at this level” refers to reads that were classified at other taxonomic levels, but not the level being examined in a given column. Fig. S1 The frequency of genera and species in the ground truth data, and in the classification results for each reference database. The total frequency is shown in the top two graphs, the middle graphs show the frequency of false positives occurring, and the bottom two graphs show the frequency of false negatives. Fig. S2 Scatterplots show the comparison between the simulated metagenomic data (ground truth, x-axis) and classified reads (y-axis) when classified using the HunRUG (A) and RefHunRUG (B) reference databases. Data are [file 42523_2022_207_MOESM1_ESM.pdf]

Additional file 1

Supplementary Table S1 *Classification status of reads for the six reference databases at various taxonomic levels.*

	Overall		Phylum		Family		Genus		Species	
Database	Classified	Unclassified	Classified	Unclassified	Classified	Unclassified	Classified	Unclassified	Classified	Unclassified
Hungate	99.95	0.05	99.94	0.06	97.99	2.01	82.58	17.42	92.69	7.31
Mini	39.85	60.15	39.40	60.60	38.44	61.56	37.21	62.79	32.20	67.80
RefSeq	50.28	49.72	49.62	50.38	48.26	51.74	46.77	53.23	43.27	56.73
RUG	45.66	54.34	45.16	54.84	42.36	57.64	27.99	72.01	43.93	56.07
RefRUG	70.09	29.91	69.53	30.47	67.05	32.95	54.69	45.31	61.53	38.47
RefHun	99.96	0.04	99.93	0.07	97.84	2.16	82.13	17.87	89.27	10.73

Classification status refers to whether the read was classified, or unclassified, regardless of accuracy. Each row denotes the six databases used to classify reads with Kraken2. The “Overall” column refers to the percentage of reads which were classified or unclassified by Kraken2 regardless of taxonomic level. Subsequent columns refer to the percentage of reads which were classified or unclassified by Kraken2 at various taxonomic levels as shown in the column headers.

Supplementary Table S2 *Classification status of reads compared to the ground truth for the six reference databases at various taxonomic levels.*

	Status	Phylum	Family	Genus	Species
Hungate	Correct	99.94	97.99	82.56	92.56
	Incorrect	0.00	0.00	0.01	0.13
	Truth unknown	0.00	1.86	16.32	0.00
	Unclassified at any level	0.05	0.05	0.05	0.05
	Unclassified at this level	0.01	0.10	1.07	7.26
Mini	Correct	38.24	35.62	32.04	20.65
	Incorrect	1.16	2.74	3.82	11.55
	Truth unknown	0.00	1.86	16.32	0.00
	Unclassified at any level	60.15	58.55	45.74	60.15
	Unclassified at this level	0.45	1.23	2.08	7.65
RefHun	Correct	99.92	97.82	81.90	88.92
	Incorrect	0.01	0.01	0.23	0.35
	Truth unknown	0.00	1.86	16.32	0.00
	Unclassified at any level	0.04	0.04	0.04	0.04
	Unclassified at this level	0.03	0.27	1.52	10.69
RefRUG	Correct	67.43	59.00	47.31	25.87
	Incorrect	2.09	7.13	5.11	35.65
	Truth unknown	0.00	1.86	16.32	0.00
	Unclassified at any level	29.91	29.51	21.63	29.91
	Unclassified at this level	0.56	2.51	9.63	8.56
RefSeq	Correct	46.13	40.93	35.97	22.74
	Incorrect	3.49	7.07	7.85	20.53
	Truth unknown	0.00	1.86	16.32	0.00
	Unclassified at any level	49.72	48.34	37.22	49.72
	Unclassified at this level	0.65	1.81	2.65	7.01
RUG	Correct	43.69	35.76	26.11	8.46
	Incorrect	1.47	5.71	1.29	35.47
	Truth unknown	0.00	1.86	16.32	0.00
	Unclassified at any level	54.35	53.80	44.41	54.35
	Unclassified at this level	0.49	2.87	11.88	1.72

The databases and detailed classification status are shown in the first column. Subsequent columns contain the percentage of reads at that taxonomic level, which had been classified by the database and had the particular classification status outlined in the first column. “Correct” and “incorrect” refer to reads which were classified correctly or incorrectly by Kraken2 using the respective database. “Truth unknown” refers to the reads that originate from genomes that do not have an assigned family or genus. “Unclassified at any level” refers to reads that were not classified to any taxonomic level. “Unclassified at this level” refers to reads which were classified at other taxonomic levels, but not the level being examined in a given column.

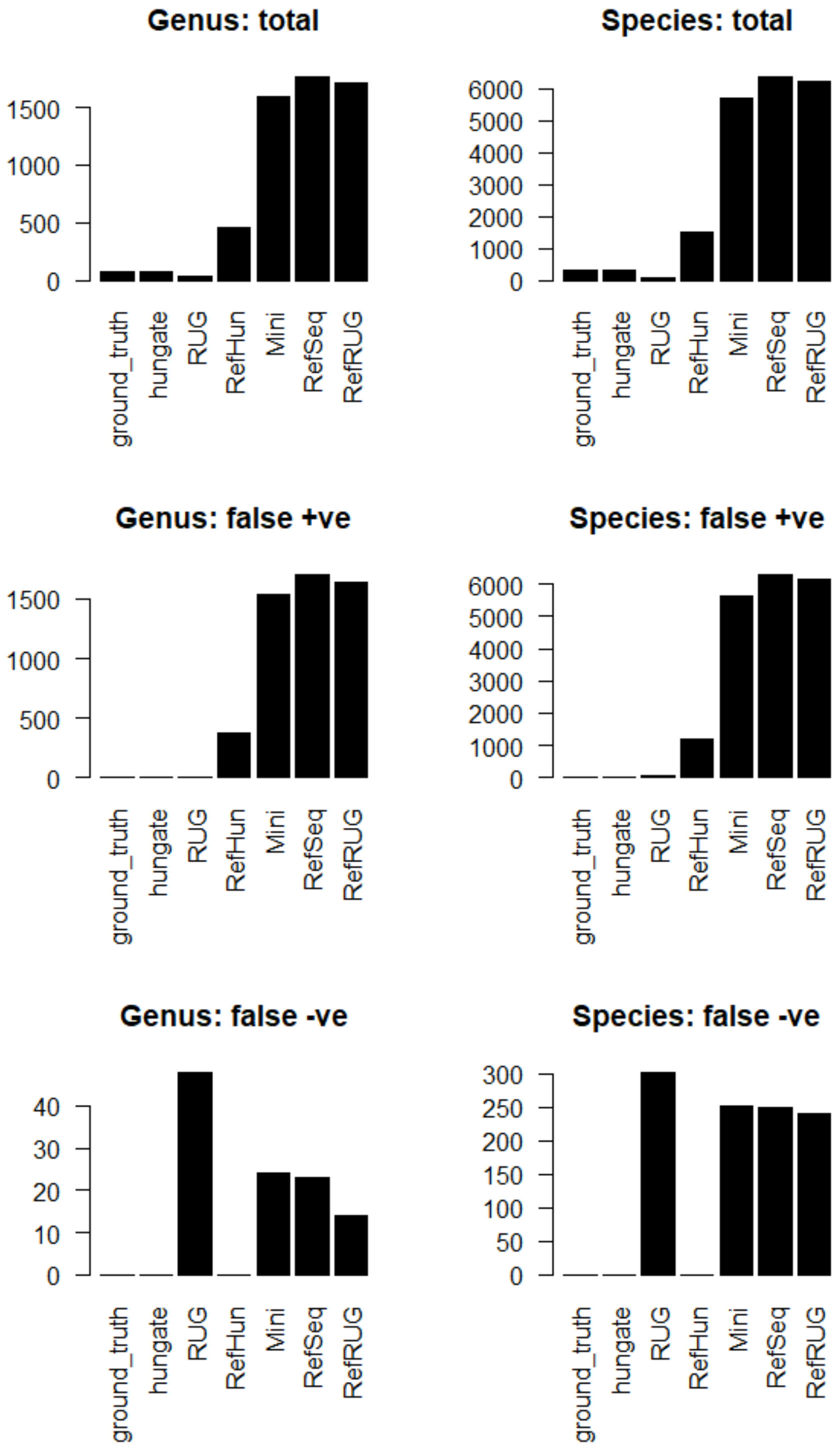

Supplementary Figure S1 The frequency of genera and species in the ground truth data, and in the classification results for each reference database. The total frequency is shown in the top two graphs, the middle graphs show the frequency of false positives occurring, and the bottom two graphs show the frequency of false negatives.

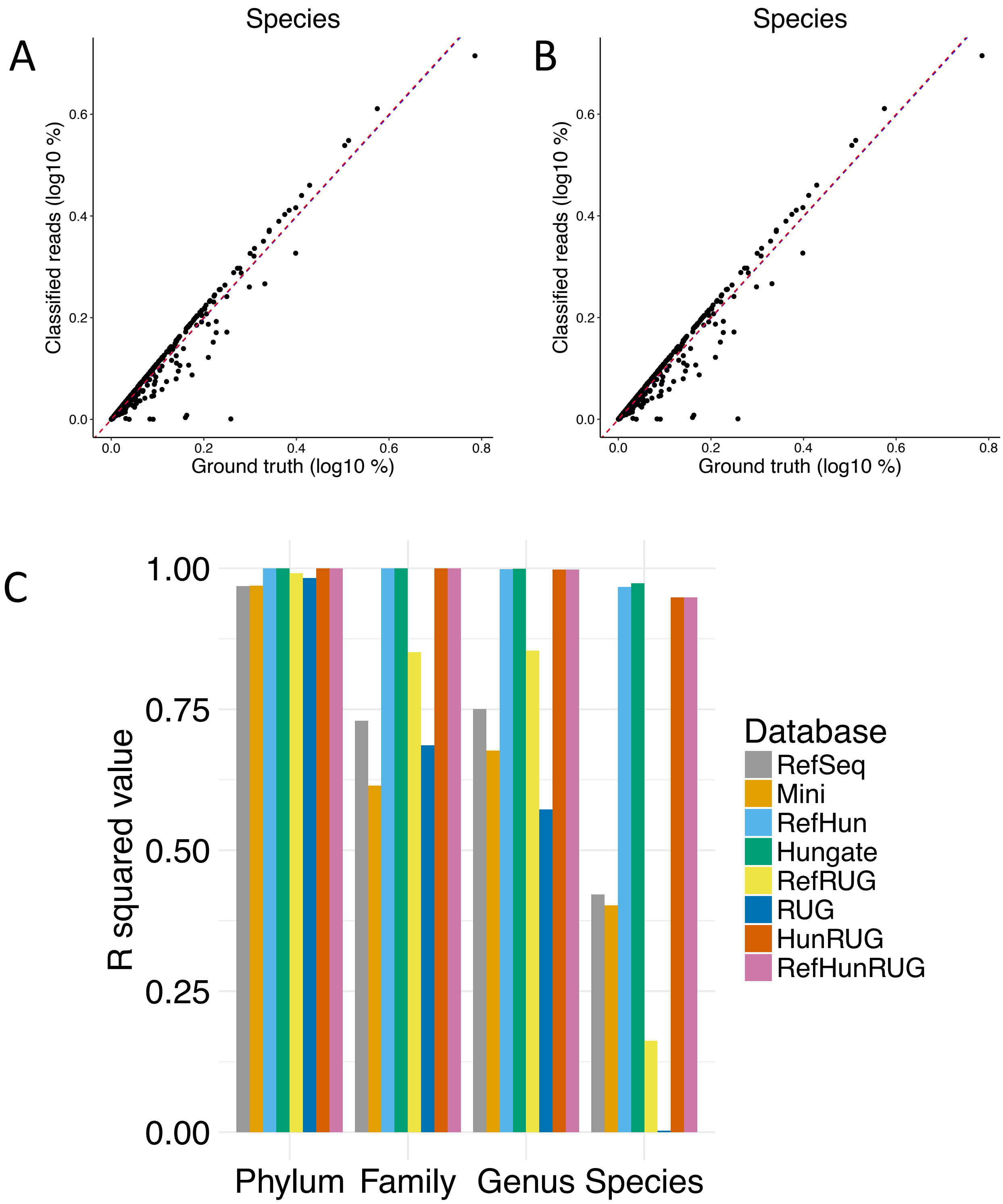

Supplementary Figure S2 Scatterplots show the comparison between the simulated metagenomic data (ground truth, x-axis) and classified reads (y-axis) when classified using the HunRUG (A) and RefHunRUG (B) reference databases. Data is plotted as a percentage of classified reads for the classified data, and a percentage of simulated reads for the ground-truth data. The data has been transformed by log10. A y=x line (shown in red) has been added to demonstrate how data points would appear on the graph if the number of ground-truth and classified reads were the same. A linear regression has been added (shown in blue) and used to calculate the R^2 statistic. The R^2 statistic is shown (C) for each reference database at the Phylum, Family, Genus and Species levels.
